# Supplementary material for: Optimizing colour for camouflage and visibility using deep learning: the effects of the environment and the observer's visual system
Source: J R Soc Interface. 2019 May 29;16(154):20190183. doi: 10.1098/rsif.2019.0183 (PMC6544896; doi:10.1098/rsif.2019.0183)
Supplement: Supplementary Information [file rsif20190183supp1.pdf]

Fennell, J. G., Talas, L., Baddeley, R. J., Cuthill, I. C., Scott-Samuel, N. E.
Optimising colour for camouflage and visibility using deep learning: the effects of the environment and the observer's visual system

Supplementary Information

Neural network architecture and parameters

Networks were trained for 500 epochs with a batch size of 128. The RMSprop optimiser was used with learning rate of 0.001 and mean squared error as loss function. The architecture of the networks is illustrated in Figure S1. All Dense layers had 768 units with ReLU activations and dropout was set to 0.5.

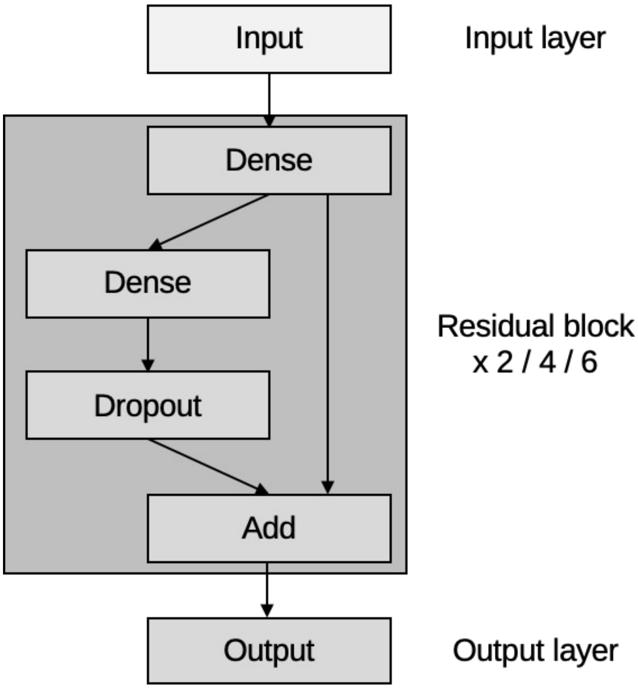

Figure 1. Schematic illustration of the residual deep neural networks used in the study.

Comparing the error between networks with different number of residual blocks

Mean validation losses were calculated for 100 bootstrapped neural networks with two, four or six residual blocks after 500 training epochs using mean squared error (Fig. S2). Statistics were calculated using random permutation tests, based on 100,000 resamples. P-values were adjusted for multiple comparisons with False Discovery Rate [1]. We found that neural networks with four residual blocks produced significantly lower error rates compared to networks with two or six residual blocks, in all four experimental conditions (Table S1).

Table S1. Comparisons of mean validation losses for networks with two, four or six residual blocks in all four experimental conditions.

**Condition**	**Comparison**	**P-value**
Temperate forest trichromat	4 vs. 2	.0093
	4 vs. 6	.0093
Temperate forest dichromat	4 vs. 2	.0012
	4 vs. 6	.0115
Semi-arid desert trichromat	4 vs. 2	< .0001
	4 vs. 6	< .0001
Semi-arid desert dichromat	4 vs. 2	.01022
	4 vs. 6	.01022

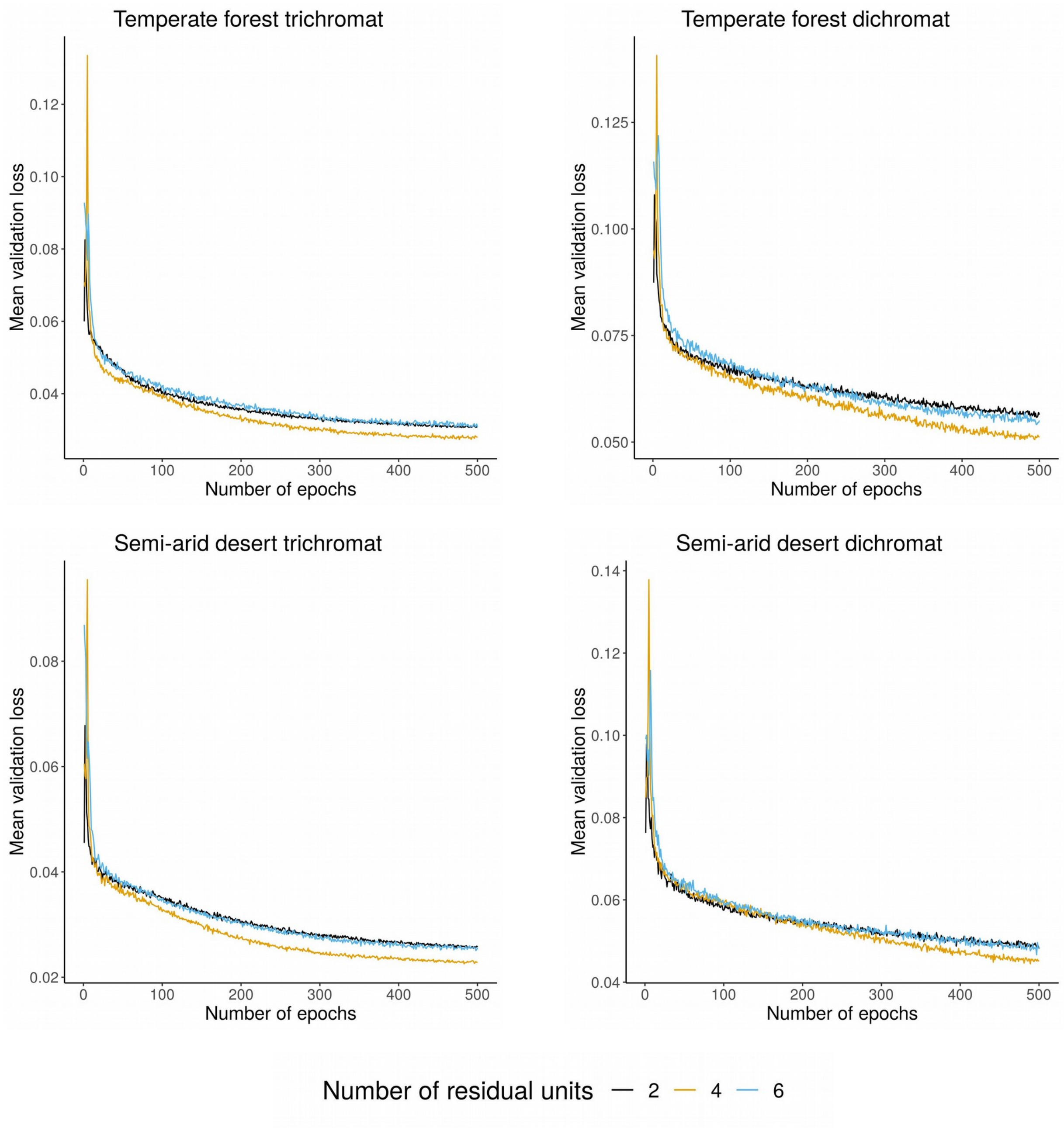

Figure S2. Mean validation losses for neural networks with two, four or six residual blocks across 500 training epochs for all four experimental conditions.

GLMM results for trichromat vs. dichromat conditions in validation experiment

The effects of trichromat vs. dichromat conditions in the validation experiment were analysed by fitting generalised linear mixed models (GLMM) with gamma distributions (log link function) using the lme4 package [2] in R [3]. Gamma distributions were chosen due to the non-normality of reaction time data (Table S6) [4]. Nested models were compared using the change in deviance on removal of a term and by the Bayesian information criterion (BIC) [5]. Participant ID was treated as a random variable within the models. If a model including a term for chromatic condition had a significantly better fit to the one without it, the effect of the chromatic condition was significant (Table S2). Pairwise post-hoc analysis revealed that in all conditions dichromat targets were harder to see than trichromat targets (Table S3). P-values were adjusted for multiple comparisons with False Discovery Rate [1].

Table S2. Comparison of GLMMs with and without the chromatic condition term.

Location	BIC (without)	BIC (with)	Δdeviance	DF	P-value
Temperate forest	-777.64	-879.4	109.55	1	< .0001
Semi-arid desert	-2168.1	-2204.7	44.443	1	< .0001

Table S3. GLMM estimates, standard error and p-values from the post-hoc analysis of dichromat vs. trichromat conditions.

Condition	Comparison	Estimate	Std. Error	P-value
Temperate forest	Dichromat Easiest vs. Trichromat Easiest	0.0628	0.0248	.0112
	Dichromat Intermediate vs. Trichromat Intermediate	0.1227	0.0248	< .0001
	Dichromat Hardest vs. Trichromat Hardest	0.2726	0.0248	< .0001
Semi-arid desert	Dichromat Easiest vs. Trichromat Easiest	0.0862	0.0213	< .0001
	Dichromat Intermediate vs. Trichromat Intermediate	0.0627	0.0213	.0032
	Dichromat Hardest vs. Trichromat Hardest	0.0898	0.0213	< .0001

GLMM results for increasing predicted difficulty in validation experiment

The effects of increasing predicted difficulty in the validation experiment were also analysed with GLMMs. If a model including a term for difficulty groups had a significantly better fit to the one without it, the effect of difficulty groups was significant (Table S4). Pairwise post-hoc analysis revealed that in all conditions progressively more difficult groups (predicted as easiest, intermediate, and hardest by the neural networks) were significantly harder to find (Table S5). P-values were adjusted for multiple comparisons with False Discovery Rate [1].

Table S4. Comparison of GLMMs with and without the difficulty groups term.

Location	Chromatic condition	BIC (without)	BIC (with)	Δdeviance	DF	P-value
Temperate forest	Trichromat	-908.72	-1068.74	174.20	2	< .0001
Temperate forest	Dichromat	272.00	37.50	248.68	2	< .0001
Semi-arid desert	Trichromat	-1176.50	-1283.00	120.68	2	< .0001
Semi-arid desert	Dichromat	-953.98	-831.31	136.85	2	< .0001

Table S5. GLMM estimates, standard error and p-values from the post-hoc analysis of difficulties in the validation experiment.

Condition	Comparison	Estimate	Std. error	P-value
Temperate forest trichromat	Easiest vs. Intermediate	0.2198	0.0203	< .0001
	Intermediate vs. Hardest	0.0423	0.0203	.0373
Temperate forest dichromat	Easiest vs. Intermediate	0.281	0.0284	< .0001
	Intermediate vs. Hardest	0.1918	0.0284	< .0001
Semi-arid desert trichromat	Easiest vs. Intermediate	0.0775	0.0194	< .0001
	Intermediate vs. Hardest	0.1371	0.0194	< .0001
Semi-arid desert dichromat	Easiest vs. Intermediate	0.1864	0.0206	< .0001
	Intermediate vs. Hardest	0.0521	0.0206	.0116

Table S6. Shapiro-Wilk normality test results of the validation experiment.

**Condition**		**W**	**P-value**
Temperate forest trichromat	Easiest	0.8186	< .0001
	Intermediate	0.8235	< .0001
	Hardest	0.866	< .0001
Temperate forest dichromat	Easiest	0.8453	< .0001
	Intermediate	0.864	< .0001
	Hardest	0.7927	< .0001
Semi-arid trichromat	Easiest	0.8623	< .0001
	Intermediate	0.8418	< .0001
	Hardest	0.7515	< .0001
Semi-arid dichromat	Easiest	0.8620	< .0001
	Intermediate	0.8785	< .0001
	Hardest	0.8594	< .0001

Table S7. Reference and measured values of projected colours using a Minolta CS-100A Luminance and Color Meter (Minolta Co., Ltd., Osaka, Japan).

Manufacturer's sRGB D65 colour values			Measured Yxy values			Description
52	53	53	4.0	0.278	0.333	Black
84	86	87	10.3	0.279	0.324	Neutral 3.5
121	121	121	21.8	0.281	0.326	Neutral 5
162	163	162	38.4	0.280	0.330	Neaural 6.5
203	204	203	60.4	0.276	0.334	Neutral 8
249	249	244	78.0	0.276	0.343	White
0	137	167	22.6	0.200	0.268	Cyan
190	87	152	17.2	0.294	0.202	Magenta
241	201	25	52.6	0.433	0.537	Yellow
174	60	61	10.6	0.501	0.326	Red
76	152	74	24.2	0.303	0.552	Green
49	68	151	8.3	0.167	0.138	Blue
230	160	45	27.4	0.465	0.497	Orange yellow
162	190	65	34.5	0.380	0.563	Yellow green
93	61	105	6.2	0.250	0.201	Purple
195	83	97	13.2	0.423	0.296	Moderate red
72	92	174	11.0	0.178	0.159	Purplish blue
222	123	51	19.2	0.304	0.439	Orange
98	191	170	29.7	0.246	0.367	Bluish green
130	129	175	17.3	0.233	0.234	Blue flower
95	109	68	10.7	0.340	0.464	Foliage
92	123	156	16.2	0.220	0.253	Blue sky
195	147	129	23.8	0.356	0.354	Light skin
117	85	72	8.5	0.374	0.362	Darkin skin

References

1. Benjamini Y, Hochberg Y. Controlling the false discovery rate: a practical and powerful approach to multiple testing. J R Stat Soc Ser B. 1995;57(1): 289–300. DOI: 10.2307/2346101

2. Bates D, Maechler M, Bolker B, Walker S. Fitting linear mixed-effects models using lme4. J Stat Softw. 2015;67(1): 1-48. DOI: 10.18637/jss.v067.i01

3. R Core Team. A language and environment for statistical computing. R Foundation for Statistical Computing, Vienna, Austria. 2018.

4. Lo S, Andrews S. To transform or not to transform: using generalized linear mixed models to analyse reaction time data. Front Psychol. 2015;01171. DOI: 10.3389/fpsyg.2015.01171

5. Schwarz GE. Estimating the dimension of a model. Ann Stat. 1978;6(2): 461-464. DOI: 10.1214/aos/1176344136
